# Decongestant use and the risk of myocardial infarction and stroke: a case-crossover study

**DOI:** 10.1038/s41598-021-83718-8

**Published:** 2021-02-18

**Authors:** Lamiae Grimaldi-Bensouda, Bernard Begaud, Jacques Benichou, Clementine Nordon, Olivia Dialla, Nicolas Morisot, Yann Hamon, Yves Cottin, Elie Serrano, Lucien Abenhaim, Emmanuel Touzé

**Affiliations:** 1 The PGRx Study Group, Paris, France; 2 Service de Pharmacologie, Hôpital Raymond Poincaré, Groupe Hospitalier Paris-Ile de France Ouest, Assistance Publique- Hôpitaux de Paris, Paris, France; 3 UFR des Sciences de La Santé, Université Paris-Saclay, Paris, France; 4 Département de Pharmacologie Médicale, Equipe “Pharmaco-Epidémiologie et Impact des Médicaments sur Les Populations”, Bordeaux Population Health Research Center INSERM U1219, Bordeaux, France; 5 Université de Bordeaux, Bordeaux, France; 6 Hôpital Universitaire de Rouen, Rouen, France; 7 INSERM U1219, Bordeaux, France; 8 GHU Paris Psychiatrie Et Neurosciences, Paris, France; 9 Laser Core Paris, Paris, France; 10 Departement de Cardiologie, Centre Hospitalo-Universitaire de Dijon, Dijon, France; 11 ORL Et Chirurgie Cervico-Faciale, Centre Hospitalo-Universitaire de Toulouse, Toulouse, France; 12 The PGRx Study Group, London, UK; 13 London School of Hygiene and Tropical Medicine and Laser Core UK, London, UK; 14 Normandie Université, Université Caen Normandie, CHU Caen Normandie, Inserm U1237, Caen, France

**Keywords:** Cardiology, Medical research, Neurology, Risk factors

## Abstract

Pharmacovigilance reports of cerebral and cardiovascular events in those who use decongestants have triggered alerts related to their use. We aimed to assess the risk of stroke and myocardial infarction (MI) associated with the use of decongestants. We conducted a nested case-crossover study of patients with incident stroke and MI identified in France between 2013 and 2016 in two systematic disease registries. Decongestant use in the three weeks preceding the event was assessed using a structured telephone interview. Conditional logistic multivariable models were used to estimate the odds of incident MI and stroke, also accounting for transient risk factors and comparing week 1 (index at-risk time window, immediately preceding the event) to week 3 (reference). Time-invariant risk factors were controlled by design. In total, 1394 patients with MI and 1403 patients with stroke, mainly 70 years old or younger, were interviewed, including 3.2% who used decongestants during the three weeks prior to the event (1.0% definite exposure in the index at-risk time window, 1.1% in the referent time window; adjusted odds ratio (aOR), 0.78; 95%CI, 0.43–1.42). Secondary analysis yielded similar results for individual events (MI/stroke). We observed no increased risk of MI or stroke for patients 70 years of age and younger without previous MI or stroke who used decongestants.

## Introduction

Sympathomimetic decongestants are used for the management of ear, nose, and throat (ENT)-related conditions. These drugs stimulate α-adrenergic receptors and are of two classes: phenylethylamines (such as epinephrine, norepinephrine, pseudoephedrine, phenylephrine, and phenylpropanolamine) and benzylimidazolines (oxymetazoline and naphazoline). Their sympathomimetic action results in vasoconstriction of nasal blood vessels, while also reducing local inflammation and mucus formation. However, their adrenergic effects are not limited to ENT areas and these drugs have systemic effects, notably on the cardiovascular system, in vivo^1,2^. Phenylpropanolamine has been reported to be associated with hemorrhagic stroke when used as an appetite suppressant^3^ or a “cold remedy”^4^ and drugs containing phenylpropanolamine were withdrawn in the US and most European countries. Isolated case reports and small case series have suggested that other nor/epinephrine-based decongestants may induce stroke^5^ or acute coronary syndrome^6,7^, leading to restrictions of use and guidelines recommending that patients with cardiovascular antecedents or risk factors not use these drugs^8^. However, sound pharmacoepidemiological studies are lacking.

This study was conducted at the request of the French medicines safety agency (Agence Nationale de Sécurité des Médicaments, ANSM) following case reports of cardiovascular events in users of decongestants in the national pharmacovigilance database^9^.

Its objective was to determine whether exposure to decongestants is associated with an increased risk of myocardial infarction (MI) or stroke.

## Methods

We conducted a nested case-crossover study using both stroke and MI registries. The case-crossover design aimed to explore associations between transient or intermittent exposure and their immediate effect on acute outcomes^10^ while allowing for complete matching of controls to each case, as each case served as its own control^11^.

### Patients

Patients included in this study had incident, not immediately fatal, stroke or MI, were living in France, and agreed to be interviewed. The stroke and MI registries were multipurpose, multicenter clinical registries that were assembled systematically (i.e. not for the specific purpose of this study) by networks of clinical centers throughout the country. Eligible strokes were identified within the “PGRx-Stroke Registry” assembled by board-certified physicians working in 63 stroke units and 15 neurology, neurosurgery, or geriatrics inpatient settings, all managing at least 50 stroke patients annually. Eligible MIs were identified in the “PGRx-Acute Coronary Syndrome (ACS) Registry” assembled by 151 cardiologists, practicing in teaching or general hospitals or outpatient settings^12–15^. Only incident cases that occurred between September 1, 2013 and June 30, 2016 and not during the months of July and August, when the use of decongestants is very low, were eligible for the study. The ANSM requested that patients aged 70 years or less be retained because exposure to decongestants in the elderly was found to be very low at the end of the first year of the study.

#### Definition of cases

Eligible strokes consisted of a first lifetime occurrence of ischemic or hemorrhagic stroke (intraparenchymal, intraventricular, or subarachnoid) confirmed by computed-tomography or magnetic resonance imaging^16^. Cases of subdural bleeding, intracranial aneurysms, cerebral vascular malformations, or severe cranial trauma that occurred < 15 days before were not considered in the present study. Eligible MIs consisted of a first lifetime occurrence of MI with at least two of the following criteria: (a) typical chest pain, (b) typical electrocardiogram abnormalities, and (c) elevated troponin levels^17^. Patients with a history of angioplasty, coronary bypass surgery, or hospitalization were excluded. The results of biological tests, imaging, and performed procedures, data on comorbid conditions and body mass index, and disease severity and risk scores (Glasgow coma scale, HAS-BLED and NIHSS) were retrieved from the registries.

### Ascertainment of exposure

Patients were interviewed to determine their exposure to decongestants, as these drugs are available over-the-counter (OTC)^18^. Eligible patients identified in the registries were rapidly approached to obtain an informed consent to be interviewed without being informed of the objective of the study. Indeed, most were interviewed for other purposes as well. They were questioned about their use of more than 300 different drugs for a variety of morbidities, including decongestants. A proxy was interviewed in cases of disabling MI or stroke or at the request of the patient. An interview guide showing images of the packaging of the most highly prescribed medications for cardiovascular, metabolic, neurological, and infectious health conditions was mailed to consenting patients or their proxy. In particular, the images displayed the packaging of the most frequently used medications for pain, flu, flu-like syndromes, and ENT-related conditions. Interviews were conducted by trained interviewers through a structured and standardized telephone interview using the previously validated Progressive Assisted Backward Active Recall (PABAR) method^19,20^. Medications reported by the patients but not listed in the interview guide were also included. The interview focused on all medications used in the six weeks preceding the event (the considered stroke or MI). Exposure to decongestants (yes/no) during each of the weeks under consideration before the event was considered to be “definite” if the patient: (i) was able to provide dates of use or no use of the drugs (ii) and/or referred to objective information indicating the use of the drugs within these time windows (such as, but not restricted to, date and/or proof of purchase or prescriptions with their date) (iii) and/or affirmed with confidence that they had used/not used the drugs in question within each of the time windows of interest. The PABAR methodology helped the participants to identify these times by linking them with their “life-events” (an event at work or a family event or other personal activity) during the period and identify whether they were sick during that event (for example, did they have a flu-like syndrome during that event? If yes, did it prompt any use of medication?). The use of decongestants was considered to be “indefinite” if the patients reported the use of decongestants during the three-week period before the event without being able to identify in which week the decongestant had been used with sufficient confidence. Exposure to decongestants was considered to be “uncertain” if the patients were unable to identify whether they were used or not during the three weeks with confidence. Finally, patients were categorized as “definitely not exposed” if no use of decongestants was reported for the three weeks.

### Analysis

The time period of interest for the study of the risk of decongestant use was the three weeks preceding the event and was split into three one-week time windows. The first week, i.e. immediately preceding the event (week -1), was considered to be the time window of potentially higher risk for the effect of the decongestants (“index at-risk time window”). The third week preceding the event (week -3) was used as the “reference” time window. The week in between the two (week -2) was not used in the main analysis. The exposure of interest was the use of any decongestant, corresponding to the following ATC codes: R01A, R01B, and R05X, regardless of the dose.

#### Outcomes

The primary outcome was MI or stroke as a composite event and the secondary outcomes were MI and stroke, separately.

#### Statistical analysis

Based on sales figures for France, we a priori estimated that 1 to 2% of the general population used decongestants each week during the fall, winter, and spring. We calculated that for a prevalence of exposure of 1 to 2% in the reference time window, a sample size of 2,700 cases would allow detection of odds ratios (ORs) of 1.76 and 1.51, respectively, with a type-I error of 5% in a one-sided test, with 80% power.

The distribution of risk factors and characteristics were compared for eligible patients from the registry with an interview vs. those who were not interviewed based on information recorded in the registry using t-tests for means and chi-square statistics for proportions. In the presented models, “definite exposure” and “definite no exposure” to decongestants were considered; “indefinite use” was not considered, as it would, by definition, concern all time windows, with no additional discordant pairs of use in the estimation of the OR. The association between decongestant use and the risk of MI or stroke was estimated using conditional logistic regression models, which provided adjusted odds ratios (aOR) and their 95% confidence intervals (95%CI). In these models, the dependent variable was the first time window (week 1) relative to the “reference” (week 3) and the main independent variable was definite exposure (Yes/No) to decongestants. All time-invariant confounders were accounted for by design (age, gender, behavioral risks, comorbidities, previous use of decongestants, and others). The following five potential time-varying confounders were a priori identified: flu, respiratory-tract infections (pneumonia or bronchitis), urinary-tract infections (cystitis, pyelonephritis, or prostatitis), dental infections, and local anesthesia (applied by a dentist, ENT specialist, or pulmonologist). Each of these confounders was included in the analysis model only if it was not found too highly associated (collinear) with exposure to decongestants, i.e., if the exposure-covariate odds ratio (ECOR) was between 0.2 and 5.0. A secondary analysis was performed for the individual type of event (MI or stroke, separately). Sensitivity analysis was conducted by type of respondent to the interview (patient alone, proxy alone, and patient & proxy), by taking four reference time windows, and by limiting the analyzed population to cases with discordant use of decongestants between the at-risk and the reference time window. Finally, exploratory analyses stratified by gender, age (categorized into < 66 vs. ≥ 66 years of age), and the presence of cardiovascular risk factors were also performed on the composite outcome. All analyses were conducted using SAS software version 9.2 (SAS Institute, Inc. NC, USA).

### Ethics approval and consent to participate

All participants in the registries provided an informed consent for their registration and those interviewed for this study consented to being interviewed. The study protocol conformed to the ethical guidelines of the 1975 Declaration of Helsinki and was approved by the ethics review committee of Paris-Ile de France III. Data collection was approved by the French data protection authority. The study was conducted under the auspices of an independent scientific committee which approved the protocol, the statistical analysis plan, and the reports to the regulatory agencies. Thirteen pharmaceutical companies commercializing vasoconstrictors participated in the funding of the study; however, the PGRx registries within which cases were identified were assembled independently and funded by multiple sources. The analyses, reports, and publications were produced independently of the pharmaceutical companies.

## Results

**Figure 1:**
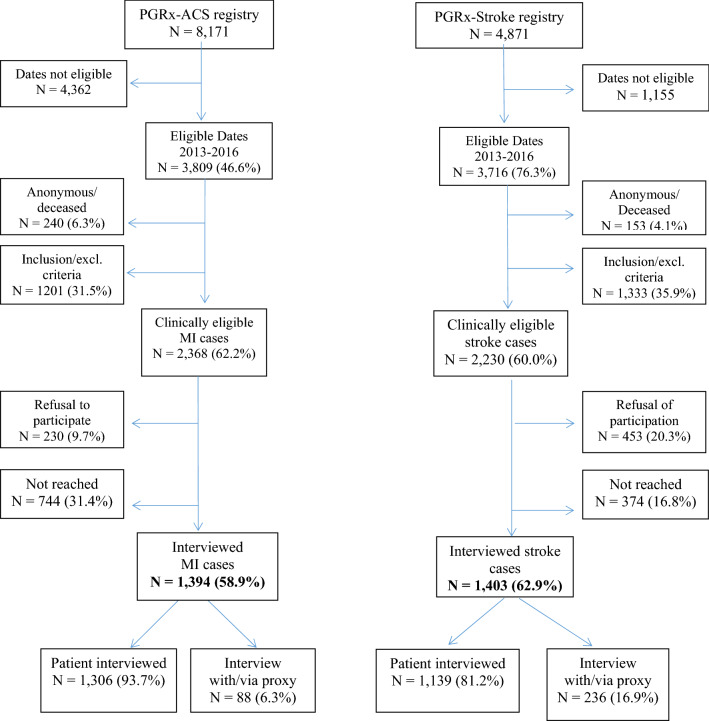
Flow chart of patient identification for the analyses conducted in the present case-crossover study. Abbreviations: ACS, acute coronary syndrome; MI, myocardial infarction.

A flow-chart presenting the process of selection of cases eligible for this study amongst the patients in the two registries is shown in Fig. 1.

In the ACS registry, 2,368 patients met the inclusion criteria for this study; among them, 1394 (58.9%) underwent a telephone interview (88 with or via their proxy). In the stroke registry, 2230 patients met the inclusion criteria for this study; among them, 1403 (62.9%) underwent a telephone interview (236 with or via their proxy). The studied population consisted of 2797 interviewed patients. Data on the interviewed and non-interviewed patients are shown in Table 1. The only notable difference between these two groups of patients was the proportion of hemorrhagic stroke among strokes (6.5% in interviewed, 12.8% in not interviewed).

**Table 1:** Characteristics of eligible patients with incident myocardial infarction or stroke (interviewed or not).

	Interviewed	Not Interviewed
	N = 2797	N = 1801
**Male gender, n (%)**	2062 (73.7%)	1234 (68.5%)
Mean age (years [SD]) at index date	58.4 [12.4]	61.0 [14.0]
Median age (years [IQR]) at index date	58.6 [50.6–64.9]	60.4 [51.0–69.0]
Median body mass index [min; max], in kg/m^2^	26.3 [14.9; 80.0]	26.2 [12.6; 76.0]
Number of cardiovascular comorbidities*, n (%)		
None	848 (30.6%)	464 (26.0%)
1	819 (29.5%)	505 (28.3%)
2	560 (20.2%)	381 (21.4%)
≥ 3	545 (19.7%)	434 (24.3%)
STEMI if MI (n, %)	820 (58.8%)	537 (63.5%)
Hemorrhagic if stroke (n, %)	91 (6.5%)	106 (12.8%)

*Hyperlipidemia, hypertension, heart failure, diabetes mellitus.

*MI* myocardial infarction, *SD* standard deviation, *STEMI* ST segment elevation myocardial infarction.

Overall, 89 (3.2%) patients reported the use of a decongestants in the three weeks preceding the event. Among them, 58 (65.2%) were classified as “definite exposure” (exposure in definite time windows), 28 (1.0% of cases) occurring during the time window immediately preceding the event (week -1) *vs*. 31 (1.1%) during the reference time window (week -3); aOR: 0.78 [95%CI 0.43–1.42] (Table 2), whereas six (0.21%) definitely used a decongestant in both time windows (week -1 and week -3). Flu occurred in only two patients (none being decongestant users) so that flu could not be included in the analysis model. The remaining four a priori identified potential time-varying confounders occurred more frequently and had ECOR with decongestant exposure between 1.0 and 5.0. Hence, the analysis model was adjusted for these confounders, as planned, using a dichotomous yes/no composite confounder variable (yes if any of the four corresponding events occurred, no otherwise).. During the intermediate time window (week -2), 26 (0.9%) patients definitely used a decongestant. The results for the secondary analysis of MI or stroke, separately, were similar (Table 2). When the analysis was limited to the patients with discordant use of decongestants, the result did not change (adjusted OR 0.77 [0.43–1.40]). Also, when four reference time windows (weeks -3, -4, -5, -6) were used in the analysis instead of one, the result was similar (adjusted OR 0.68 [0.42–1.12]). The results of the sensitivity analysis by type of respondent to the interview show no significant association between exposure to the use of decongestants and the occurrence of MI and/or stroke (as a composite outcome) relative to no use of decongestants. This relationship (no association) held in both univariate and adjusted multivariate models (Interviews conducted with patient alone: crude OR 0.77 [0.41–1.48]; adjusted OR 0.78 [0.41–1.48], conducted with proxy alone: crude OR 0.50 [95% CI 0.05–5.51]; adjusted OR 0.52 [95% CI 0.05–5.94], conducted with patient and proxy: crude OR 1.00 [95% CI 0.06–15.99]; adjusted OR 1.00 [95% CI 0.06–15.99]). The results of the exploratory analysis stratified by gender, age categories (< 66 y/ ≥ 66 y), and prevalence of cardiovascular risk factors (≤ 1/ > 1) are also presented in Table 2. The median duration of definite use of decongestants in the study was four days (IQR: 3–7) and only 6 (11.3%) cases had definite exposure during both week -1 and week -3.

**Table 2:** Association between definite exposure to decongestants and the odds of incident myocardial infarction or stroke, pooled or separated and by gender and age.

	Patients exposed in the index at-risk time window (week -1)	Patients exposed in the referent time window (week -3)	Crude conditional OR [95%CI]	Adjusted* conditional OR [95%CI]
Exposure to decongestants				
All events (Stroke + MI)	N = 2797	N = 2797		
Definite exposure n, %	28 (1.0%)	31 (1.1%)	0.77 [0.43–1.39]	0.78 [0.43–1.42]
Stroke only	n = 1403^‡^	n = 1403^‡^		
Definite Exposure n, %	20 (1.4%)	19 (1.4%)	0.90 [0.43–1.87]	0.89 [0.42–1.85]
Ischemic stroke **	n = 1322^‡^	n = 1322^‡^		
Definite exposure n, %	18 (1.4%)	17 (1.3%)	0.89 [0.42–1.91]	0.88 [0.41–1.88]
Hemorrhagic Stroke only **	n = 91^‡^	n = 91^‡^		
Definite exposure n, %	2 (2.2%)	2 (2.2%)	1.00 [0.06–15.99]	1.00 [0.06–15.99]
Myocardial infarction only	n = 1394	n = 1394		
Definite exposure	8 (0.6%)	12 (0.9%)	0.60 [0.22–1.65]	0.66 [0.24–1.85]
Stratified analyses on all events (Stroke + MI) ***				
*Females*	n = 735	n = 735		
Definite exposure n, %	9 (1.2%)	11 (1.5%)	0.59 [0.20–1.78]	0.59 [0.20–1.79]
< *66 years*	n = 2220	n = 2220		
Definite Exposure n, %	23 (1.0%)	28 (1.3%)	0.69 [0.36–1.32]	0.69 [0.36–1.33]
≥ *66 years*	n = 577	n = 577		
Definite exposure n, %	5 (0.9%)	3 (0.5%)	1.33 [0.30–5.96]	1.48 [0.33–6.72]
*Cardiovascular risk factors (*≤ *1)*	n = 1667	n = 1667		
Definite exposure n, %	20 (1.2%)	20 (1.2%)	0.91 [0.44–1.85]	0.96 [0.47–1.98]
*Cardiovascular risk factors (*> *1)*	n = 1105	n = 1105		
Definite exposure n, %	7 (0.6%)	11 (1.0%)	0.56 [0.19–1.66]	0.55 [0.18–1.63]

*Adjusted odds ratio estimated using conditional logistic regression models including potential time-varying confounders (respiratory-tract infections (pneumonia or bronchitis), urinary-tract infections (cystitis, pyelonephritis, or prostatitis), dental infections, and local anesthesia (applied by a dentist, ENT specialist, or pulmonologist) included in one dichotomous yes/no composite confounder variable (yes if any of the four corresponding events occurred, no otherwise), with “No exposure” as the reference.

**Including 10 cases with ischemic and hemorrhagic stroke.

***Exploratory analysis.

*CI* confidence interval, *MI* myocardial infarction; *OR* odds ratio.

## Discussion

The present study is the first large epidemiological study to investigate the association between the use of decongestants presently marketed worldwide and MI and all strokes (ischemic and hemorrhagic). This study was based on a large sample of almost 2800 cases of fully clinically documented stroke or MI stemming from 200 clinical centers spread throughout France, including half of the neurovascular units of the country. We did not observe an association between exposure to decongestants and the occurrence of MI or stroke, either used as a composite outcome or secondarily studied separately. Exploratory analysis stratified by event (stroke and MI alone) was not performed due to the low level of the use of decongestants, which led to small sample sizes for the exposed at-risk period.

Nesting the study within systematic disease registries also significantly reduced the possibility of selection bias. The case-crossover design has the advantage of eliminating the effect of a large number of potential known or unknown confounders, as patients served as their own controls.

This study had several limitations. The first was the paucity of data for patients over 70 years of age due to the local restriction of the use of decongestants in this population, which prompted a restriction in their recruitment. Thus, these results cannot be generalized to elderly patients over 70 years of age. In addition, patients who died rapidly after their event were not identified or interviewed in time.

Interviewed participants were relatively similar to those that were not interviewed, although they were slightly younger (by 2.6 years on average) and less frequently suffered from a hemorrhagic stroke (Table 1). This is likely explained by the higher severity of hemorrhagic stroke, precluding interview of many of these patients. The secondary analysis of ischemic strokes separately showed results very close to those of the main analysis; we also observed no difference in hemorrhagic strokes but the sample size of subjects exposed to decongestants in this subgroup was quite small (Table 2). The exploration of the effect of decongestants on hemorrhagic stroke alone was not feasible in this study due to the small size of the subgroup. Addressing this question remains a challenge, especially given that several case reports were based on hemorrhagic stroke^5,21^. The observed rate of exposure (c. 1% definite, 1% indefinite each week) was in the expected range (1 to 2% per week) on which the sample size of the study was based (based on sales figures) for the study of the composite outcome (strokes and MI). The study reached the planned sample size and statistical power, but such low exposure levels limited the possible number of validation analyses and explorations that could be conducted. Another concern for the efficiency of this study could be the overmatching. According to MacLure and Mittleman^11^, self-matching is equivalent to overmatching only if there is little or no confounding by unmeasured constant characteristics, nor much residual confounding by poorly measured constant characteristics. This rarely happens if exposure has both an acute and a chronic effect, because past exposure is usually highly correlated with recent exposure. This is true for the exposure of interest in our study: Individuals currently exposed to decongestants are those who were also exposed in the past. Indeed, there was 96.2% agreement between the at-risk time window and the −3 to −6 week time window among the 2797 patients (1% were exposed in both time windows and 95.2% were not exposed in either time windows). When the agreement of exposure is compared between the at-risk time window and the remote past (prior to -6 weeks), the percentage of agreement is 70.7% (0.7% exposure), which is still high.

Interview-based studies are always suspect of “recall bias”. If one remembers the preceding week better than what happened three weeks ago (referent period), i.e., differential misclassification, it would be biased against the drug, increasing the estimation of the OR. However, cases in this study tended to report *less* rather than more exposure in the week immediately before the event, which does not support such a bias (although there are examples to the contrary)^22,23^.

The case-crossover design relies on the assumption that the disease has an acute onset and that the exposures of interest are transient. The former assumption is by definition met for incident stroke and MI. The short median duration of use of decongestants (median 4 days) and low frequency of use over the three-week period studied (11.3%) shows that the second assumption was generally also met. Although the case-crossover design excludes confounding by permanent risk factors, whether they are known or unknown, it is not immune to confounding associated with transient risk factors that occur at the time of the exposure of interest. We were able to document and control for certain known transient risk factors and potential confounders, such as flu or flu-like syndromes, respiratory tract infections, other infections, and local anesthesia (contra-indication to decongestant use) reported in the literature^24–26^, which can be both linked to decongestant use and the risk of stroke or MI. Conversely, we did not document other transient events considered to be risk factors for MI or stroke^27^, such as heavy meals^28^, anger ^29^, other negative emotions^30^, air pollution peaks^31^, or disasters^32,33^, some of which may be positively or negatively associated with decongestant use. Although we do not believe that these relatively weak risk factors, which act in opposite directions for some, significantly affected the results, it is impossible to completely exclude residual confounding associated with unmeasured, transient factors. It is possible that patient self-selection to exposure occurred, in that patients with a cardiovascular risk would not expose themselves to a decongestant, a phenomenon coined as a “depletion of susceptibles” effect^34^, limiting the ability to identify the effects of decongestants in people with risk factors or previous experience of cardiovascular diseases, as proposed in several case reports^35–37^. This may be reflected by the lower OR of stroke or MI in most of our results, especially for patients with a history of cardiovascular risk factors (Table 2), although none of these ORs were significantly different from 1.

## Conclusions

Our study observed no association between decongestant use and the occurrence of stroke or MI. Further research is recommended on elderly patients and those with hemorrhagic stroke, a difficult task given the low use of these drugs by the elderly and the necessity to interview patients for the study of OTC drugs.

## Data Availability

The datasets generated and/or analyzed during the current study are not publicly available but are available from the corresponding author. Contact: lamiae.grimaldi@aphp.fr
